# Medical residents’ mental distress in the COVID-19 pandemic: An urgent need for mental health care

**DOI:** 10.1371/journal.pone.0266228

**Published:** 2022-03-31

**Authors:** Amanda Steil, Ana Bresser Pereira Tokeshi, Luca Silveira Bernardo, Geraldo Pio da Silva Neto, Ronald Ferreira Davi, Amanda Ferreira Santa Bárbara, Vitor S. Mendonca, Thiago M. Fidalgo, Aécio Flávio Teixeira Gois

**Affiliations:** 1 Disciplina de Medicina de Urgência and Medicina Baseada em Evidências, Departamento de Medicina, Universidade Federal de São Paulo, São Paulo, Brazil; 2 Departamento de Psiquiatria, Universidade Federal de São Paulo, São Paulo, Brazil; 3 School of Medicine, University of São Paulo, São Paulo, Brazil; 4 Fellow of American College of Physicians, Philadelphia, Pennsylvania, United States of America; University of the Witwatersrand, SOUTH AFRICA

## Abstract

**Purpose:**

Medical residents’ mental health is currently an issue of concern for medical educators worldwide. The COVID-19 pandemic has raised the greatest concerns given the psychological effects of this scenario on medical residents on the frontlines of the pandemic. To assess the psychological impact of the COVID-19 pandemic on physicians in residency training, the collective symptoms of burnout, depression and anxiety are used to identify the residents’ beliefs and clinical practices related to COVID-19 patients and their behaviors concerning disease prevention.

**Method:**

This observational study involved 3071 medical residents from all regions of Brazil. An online questionnaire assessed the presence of burnout using the Oldenburg Burnout Inventory, depressive symptoms using the Patient Health Questionaire-9, anxiety symptoms using the Generalized Anxiety Disorder-7, and COVID-19 Impact Questions to assess the residents’ beliefs and clinical practices related to COVID-19 patients. Exploratory analyses, logistic regression and multinomial regression analysis were performed in this investigation.

**Results:**

Moderate and severe depressive symptoms were the most common (67.7%) followed by anxiety symptoms (52.8%) and burnout (48.6%). The difference between residents with or without contact with COVID-19 patients was significant increased when analyzing different aspects of clinical practice, behavior, substance use and mental health.

**Conclusions:**

These results suggest an increase in depression and anxiety symptoms among medical residents dealing with COVID-19, upstaging previous concerns about medical residents’ mental health. The prevalence of burnout is similar to that of a nonpandemic scenario. Considering the severity of the pandemic scenario and the overburden of healthcare services, medical residents’ mental health deserves special care.

## Introduction

Around the world, physicians of various specialties were called up to work in the fight against the COVID-19 [1, 2]. Among these professionals, resident physicians were reallocated from their initial rotations to the emergency department, intensive care units and COVID-19 wards to supply the need of medical personnel [3, 4]. This is a complex decision that has been discussed due to critical shortages of personal protective equipment (PPE) and a paucity of clinical experience in residents of all specialties [5].

In the last decade there has been an alarming increase in mental illness and suicides in the medical community. This presses for the need to examine psychological health in new light [6, 7]. Another review and metanalysis analyzed the psychological effects of emerging virus outbreaks on healthcare workers and found that staff in high-risk areas exhibited increased levels of acute or posttraumatic stress and psychological distress [8]. Risk factors included younger age and less experience, two aspects that may be related to medical residents, a group that clearly needs guidance from more experienced staff to help them achieve the required knowledge and ethical maturity to deal with the difficulties and feelings inherent of this period, which can be an extremely rich learning experience [9].

The objective of this research is to determine the frequency of depressive symptoms, anxiety, and burnout of the pandemic on Brazilian medical residents in training. Due to findings, the authors proposed methods of prevention and coping mechanisms to deal with the issues.

## Methods

An observational study was conducted according to Strengthening the Reporting of Observational Studies in Epidemiology (STROBE) parameters to assess the psychological association of COVID-19 pandemics on medical residents from Brazil. This study used a convenience sample from an anonymous online survey advertised on social media with a link invitation that could be accessed voluntarily, and distributed by e-mails for residency committees, residency associations and universities’ hospitals throughout the country. Given that a convenience sample was used, no calculation of sample size was performed. Advertisement of the research was performed using good practices guidelines [10]. There was no financial support for the volunteers.

The survey was available during the month of April 2020. This investigation collected sociodemographic information and used the Oldenburg Burnout Inventory (OLBI) to measure burnout, the Patient Health Questionnaire (PHQ-9) to measure depression, and the General Anxiety Disorders (GAD-7) to measure generalized anxiety disorder [11, 12]. All three scales were previously adapted and validated for use in the Brazilian context and population. The authors developed a COVID-19 Impact Questions to assess the residents’ beliefs and clinical practices related to COVID-19 patients, their behaviors concerning the disease prevention and their substance use after the beginning of the pandemic. All fields were marked as mandatory, so a participant could move forward only after answering all questions. Therefore, all included participants completed the entire questionnaire, so no data were missing. The protocol was reviewed and approved by the Universidade Federal de São Paulo, Brazil (UNIFESP) Research Ethics Committee (Protocol#3,943,348 on March 20, 2020). The participants’ inclusion criteria were to be a medical resident in training.

First, this investigation conducted exploratory analyses using basic contingency tables with Chi-square tests. The residents’ sociodemographic variables, characteristics of the residence program, clinical practice and beliefs of COVID-19, COVID-19-related behaviors, substance use and mental health symptoms were described. The multinomial regression and two logistic regression models included the binary variables “burnout” and “anxiety” as dependent variables. Burnout was defined as positive if the total score on OLBI was 21; anxiety was defined as positive if the total score on GAD-7 was 10 or greater. For the multinomial regression model, the variable “depression” was used with its three categories (no depression or mild depression (PHQ-9 score of 9 or less), moderate (PHQ-9 score between 10 and 14) and severe depression (PHQ-9 score of 15 or higher)) as the dependent variable. The independent variables for all the regressions were having contact with COVID-19 patients, feeling prepared to treat these patients, avoidance of seeing these patients, proper support from supervisors, working in a wing with a high risk of contamination with COVID-19, the belief personal protection equipment (PPE) is not efficacious, having access to PPE, fear of getting COVID-19 and transmitting it to significant others and impairment of personal relationships since the beginning of the pandemic. All analyses were controlled by gender, specialty (clinical specialties, surgical specialties and diagnostic and therapeutic support (i.e., pathology, radiotherapy and nuclear medicine)) and nature of the hospital (public or private).

Analyses were performed using Stata 16.0 (StataCorp LLC, Texas, USA) with estimation of 95% confidence intervals (95% CI). The results are presented as proportions, crude and adjusted odds ratios (cOR and aORs) and crude and adjusted relative risk ratio (cRRR and aRRR).

## Results

Our sample comprised 3071 residents from throughout the country. The response rate represents approximately 10% of medical residents from Brazil. Most were women, white, residents of clinical specialties held in programs provided by a university and living alone. The mean age of the sample was of 28.0 years old (SD: 3.2), and all 26 Brazilian states and the federal district were represented. Moderate and severe depressive symptoms were the most common (67.7%) followed by anxiety symptoms (52.8%) and burnout (48.6%). The difference between residents with or without contact with COVID-19 patients was significant when analyzing different aspects of clinical practice, behavior, substance use and mental health (Table 1).

**Table 1:** Descriptive statistics of 3071 residents from Brazil, 2020.

	Contact with Covid-19						
	Yes n = 1926		No n = 1145		Total n = 3071		
	n	%	n	%	n	%	p value
Gender							>0.05
Male	472	24.5	288	25.2	760	24.7	
Female	1454	75.5	857	74.8	2311	75.3	
Ethnicity							>0.05
White	1508	78.3	878	76.7	2386	77.7	
Non-white	418	21.7	267	23.3	665	22.3	
Specialty							**<0.01**
Clinical specialties	1326	68.8	683	59.7	2009	65.4	
Surgical specialties	534	27.7	413	36.1	947	30.8	
Diagnostic and therapeutic support	66	3.4	49	4.3	115	3.7	
Hospital nature							**0.01**
Public	1623	84.3	1005	87.8	2628	85.6	
Private	303	15.7	140	12.2	443	14.4	
Program provided by a university	1285	66.7	816	71.3	2101	68.4	**0.01**
Live alone	743	38.6	415	36.2	1158	37.7	>0.05
Covid-19 clinical practice and beliefs							
The hospital is prepared to treat patients with Covid-19	887	46.1	491	42.9	1378	44.9	**<0.01**
Feel prepared to treat patients of Covid-19	904	46.9	415	36.2	1319	43.0	**<0.01**
Supervisors do not provide the necessary support for the treatment of Covid-19’s patients	739	38.3	474	41.3	1213	39.4	>0.05
Avoidance of seeing patients with confirmed or suspected cases of Covid-19	558	29.0	483	42.2	1041	33.9	**<0.01**
Work in a wing with high risk of contamination with Covid-19	1606	83.4	599	52.3	2205	71.8	**<0.01**
Feeling of safeness during work	491	25.5	324	28.3	815	26.5	>0.05
Personal protection equipment is not efficacious to keep me safe	1240	64.3	750	65.5	1990	64.7	>0.05
Hospital provides personal protection equipment	672	34.9	502	43.8	1174	38.2	**<0.01**
I feel I will be a better professional after the pandemic	1287	66.8	667	58.3	1954	63.6	**<0.01**
Covid-19 behaviors							
Read news about the pandemic all the time	1574	81.7	917	80.1	81.1	2491	>0.05
Afraid of getting Covid-19 and transmitting it to my significant ones	1790	92.9	1050	91.7	2840	92.5	>0.05
Felt physical reactions, such as sweating, difficulty breathing, nausea or flutter	672	34.9	321	28.0	993	32.3	**<0.01**
Afraid of keeping habits of excessive disinfection after the pandemic	954	49.5	503	43.9	1457	47.4	**0.01**
Personal relationships impaired since the pandemic	1494	77.6	809	70.7	2303	75.0	**<0.01**
Substance use after pandemic							
Increase of alcohol use	473	24.6	248	21.7	721	23.5	>0.05
Increase of marijuana use	54	2.8	18	1.6	72	2.3	**0.03**
Increase of tobacco use	85	4.4	31	2.7	116	3.8	**<0.01**
Increase of stimulants (cocaine, amphetamines) use	80	4.2	42	3.7	122	4.0	>0.05
Mental Health							
Depression							**<0.01**
absent or mild	571	29.6	421	36.8	992	32.3	
moderate	517	26.8	324	28.3	841	27.4	
severe	838	43.5	400	34.9	1238	40.3	
Anxiety							**<0.01**
absent or mild	861	44.7	587	51.3	1448	47.2	
moderate or severe	1065	55.3	558	48.7	1623	52.8	
Burnout							**<0.01**
absent or mild	921	47.8	658	57.5	1579	51.4	
moderate or severe	487	42.5	1005	52.2	1492	48.6	

In terms of clinical practice, residents who had contact with COVID-19 patients were more likely to feel that the residents and the hospital were prepared to treat patients with this disease, and residents felt that they will better professionals after the pandemic. Residents also had significantly more physical reactions to the pandemic, were worried about keeping excessive hygiene habits after the pandemic and felt their personal relationships were impaired due to the pandemic. These residents also experienced more anxiety, depression and burnout and declared an increase in marijuana and tobacco use (Table 1).

These aspects were related to burnout: avoidance of seeing patients with confirmed or suspected cases of COVID-19 (aOR = 1.30; 1.11–1.53), the lack of supervisor support for the treatment of COVID-19 patients (aOR = 1.46; 1.24–1.71), working in a wing with a high risk of contamination (aOR = 1.41; 1.19–1.69), the belief that PPE is not efficacious (aOR = 1.38; 1.16–1.63), fear of getting COVID-19 and transmitting it to significant others (aOR = 1.63; 1.22–2.20) and having personal relationships impaired since the pandemic (aOR = 1.61; 1.35–1.91) (Table 2).

**Table 2:** Logistic regression model of burnout and anxiety symptoms among 3071 residents from Brazil, 2020.

	Burnout				Anxiety			
	cOR^a^	aOR^b^	95%CI^c^	p value	cOR	aOR	95%CI	p value
Gender (ref: male)	1.21	1.09	0.92–1.30	0.34	2.00	1.64	1.37–1.96	**<0.01**
Specialty								
Diagnostic and therapeutic support	ref				ref			
Clinical specialties	1.37	1.39	0.93–2.01	0.10	1.01	1.04	0.69–1.56	0.86
Surgical specialties	1.11	1.04	0.70–1.57	0.83	0.69	0.64	0.42–0.97	0.03
Hospital nature (ref: private)	0.98	1.09	0.88–1.34	0.44	0.90	1.07	0.86–1.33	0.54
Have contact with Covid-19 patients	1.47	1.33	1.13–1.57	**<0.01**	1.30	1.17	0.99–1.38	0.07
Feel prepared to treat patients of Covid-19	0.88	0.97	0.83–1.14	0.75	0.56	0.61	0.52–0.72	**<0.01**
Avoidance of seeing patients with confirmed or suspected cases of Covid-19	1.36	1.30	1.11–1.53	**<0.01**	1.56	1.32	1.12–1.56	**<0.01**
Supervisors do not provide the necessary support for the treatment of Covid-19’s patients	1.53	1.46	1.24–1.71	**<0.01**	1.43	1.32	1.12–1.56	**<0.01**
Work in a wing with high risk of contamination with Covid-19	2.55	1.41	1.19–1.69	**<0.01**	1.59	1.48	1.23–1.77	**<0.01**
Personal protection equipment is not efficacious to keep me safe	0.54	1.38	1.16–1.63	**<0.01**	1.75	1.48	1.24–1.76	**<0.01**
Hospital provides personal protection equipment	1.62	0.96	0.81–1.14	0.68	0.81	0.96	0.80–1.15	0.65
Afraid of getting Covid-19 and transmitting it to my significant ones	2.03	1.63	1.22–2.20	**<0.01**	3.49	2.66	1.93–3.67	**<0.01**
Personal relationships impaired since the pandemic	1.75	1.61	1.35–1.91	**<0.01**	2.65	2.56	2.14–3.06	**<0.01**

^a^ crude odds ratio;

^b^ adjusted odds ratio;

^c^ 95% confidence interval

Women were more likely to have anxiety symptoms than men (aOR = 1.43; 1.37–1.96). Avoidance of seeing patients with confirmed or suspected cases of COVID-19 (aOR = 1.32; 1.12–1.56), failure of supervisor support for the treatment of COVID-19 patients (aOR = 1.32; 1.12–1.56), working in a wing with high risk of contamination (aOR = 1.48; 1.23–1.77), the belief that personal protection equipment is not efficacious (aOR = 1.48; 1.23–1.77) and fear of getting COVID-19, transmitting it to significant others (aOR = 2.66; 1.93–3.67) and having personal relationships impaired since the pandemic (aOR = 2.56; 2.14–3.06) increased the odds of developing anxiety symptoms. A reduction in the odds of developing anxiety symptoms was found in residents of surgical specialties (aOR: 0.64; 0.42–0.97) and residents who felt prepared to treat COVID-19 patients (aOR: 0.61; 0.52–0.72).

According to Table 3 results, women exhibited an increase in the odds of having moderate (aRRR = 1.61, 1.30–2.00) and severe (aRRR = 2.13; 1.72–2.64) depression symptoms. An increase in the odds of developing moderate and severe depression was associated with avoidance of seeing patients with confirmed or suspected cases of COVID-19 (moderate: aRRR = 1.36; 1.10–1.68; severe: aRRR = 1.43; 1.16–1.75), working in a wing with a high risk of contamination (moderate: aRRR = 1.38; 1.10–1.72; severe: aRRR = 1.53; 1.23–1.90), the belief that personal protection equipment is not efficacious (moderate: aRRR = 1.38; 1.11–1.71; severe: aRRR = 1.42; 1.16–1.75), fear of getting COVID-19 and transmitting it to significant others (moderate: aRRR = 1.56; 1.10–2.21; severe: aRRR = 2.22; 1.54–3.20) and having personal relationships impaired since the pandemic (moderate: aRRR = 2.18; 1.176–2.70; severe: aRRR = 4.07; 3.27–5.07). Feeling that supervisors did not provide the necessary support for the treatment of COVID-19 patients only increased the odds of having symptoms of severe depression (aRRR = 1.81; 1.48–2.22).

**Table 3:** Multinomial logistic regression model of depressive symptoms among 3071 residents from Brazil, 2020.

	Depression^d^								
	Mild	Moderate				Severe			
	Ref	cRRR^a^	aRRR^b^	95%CI^c^	p value	cRRR	aRRR	95%CI	p value
Gender (ref: male)		1.80	1.61	1.30–2.00	**<0.01**	2.53	2.13	1.72–2.64	**<0.01**
Specialty									
Diagnostic and therapeutic support		ref				ref			
Clinical specialties		0.93	0.96	0.58–1.59	0.88	1.22	1.28	0.78–2.12	0.33
Surgical specialties		0.70	0.66	0.40–1.11	0.12	0.76	0.67	0.40–1.12	0.13
Hospital nature (ref: private)		0.95	1.10	0.83–1.43	0.53	0.80	0.99	0.76–1.30	0.96
Have contact with Covid-19 patients		1.18	1.09	0.88–1.34	0.44	1.54	1.42	1.16–1.75	**<0.01**
Feel prepared to treat patients of Covid-19		0.66	0.72	0.59–0.89	**<0.01**	0.50	0.59	0.48–0.72	**<0.01**
Avoidance of seeing patients with confirmed or suspected cases of Covid-19		1.51	1.36	1.10–1.68	**<0.01**	1.68	1.43	1.17–1.75	**<0.01**
Supervisors do not provide the necessary support for the treatment of Covid-19’s patients		1.26	1.20	0.97–1.5	0.09	1.92	1.81	1.48–2.22	**<0.01**
Work in a wing with high risk of contamination with Covid-19		1.42	1.38	1.10–1.72	**<0.01**	1.74	1.53	1.23–1.90	**<0.01**
Personal protection equipment is not efficacious to keep me safe		1.55	1.38	1.11–1.71	**<0.01**	1.96	1.42	1.16–1.75	**<0.01**
Hospital provides personal protection equipment		0.83	0.92	0.74–1.16	0.50	0.64	0.74	0.60–0.92	**<0.01**
Afraid of getting Covid-19 and transmitting it to my significant ones		2.02	1.56	1.10–2.21	**0.01**	3.16	2.22	1.54–3.20	**<0.01**
Personal relationships impaired since the pandemic		2.18	2.18	1.76–2.70	**<0.01**	3.90	4.07	3.27–5.07	**<0.01**

^a^ crude relative risk ratio;

^b^ adjusted relative risk ratio;

^c^ 95% confidence interval

Individuals who felt they were prepared to treat COVID-19 patients presented a decrease in the odds of having moderate (aRRR = 0.72; 0.59–0.89) and severe (aRRR = 0.59; 0.48–0.72) depression. The provision of personal protection equipment by the hospital also led to a decrease in the odds of presenting severe depression (aRRR = 0.74; 0.60–0.92).

## Discussion

Three main findings emerge from this study with medical residents from a convenience nationwide sample in Brazil: i) depression, anxiety and burnout are frequent in medical residents working in the COVID-19 pandemic; ii) exposure to COVID-19 patients may be related to changes in clinical practice, personal behavior, and substance use; iii) different fears, confidences and beliefs are related to the development of mental disorder symptoms.

Medical residents’ mental health is already a topic that has worried medical educators worldwide even before the pandemic scenario emerged. A global systematic review and meta-analysis from 2015 reported that the prevalence of depression or depressive symptoms among resident physicians was 28.8%, ranging from 20.9% to 43.2% [13]. In 2014, the prevalence rates for anxiety, depression and burnout were 41.3%, 21.6% and 58.4%, respectively, among Brazilian residents. Therefore, although the prevalence of burnout identified is similar to that of nonpandemic scenario, symptoms of anxiety are two-fold increased and symptoms of depression exhibit an approximately three-fold increase in the COVID-19 pandemic scenario [14]. These findings are similar to those of a survey of 1,257 health care workers in contact with COVID-19 patients in China, which reported high rates of depression (50.4%), anxiety (44.6%), and distress 71.5% using the same instruments as those used in this study [15]. Mental distress was also reported in Italy where only 20% to 25% of healthcare workers declared to feel psychologically safe and only 48% reported having access to mental health care [16]. A study published in Brazil noticed that the pandemic had a negative impact on the mental health of medical students and newly graduated doctors. They felt fear of acquiring and transmitting COVID-19 and also described psychiatric illness and use of psychotropic drugs. [17]

Given our findings in relation to different behaviors between medical residents exposed to patients infected or suspected of infection with COVID-19 and the factors correlated with the development of mental disorder symptoms, we propose the following interventions to prevent mental disease in these group.

### Training and supervision

During the severe acute respiratory syndrome (SARS) outbreak, physicians who had treated only one patient with the syndrome were more likely to feel unprepared compared with those who had treated two or more patients. This finding indicates physicians gain more confidence as they see more patients with the disease [18]. This notion may explain the differences between residents without and with contact with COVID-19 patients and why the latter were more likely to feel that they and the hospital was prepared to treat patients with this disease and that they would be better professionals after the pandemic.

Contact with these patients alone however does not address all mental health issues. This investigation also found that when residents feel supervisors do not provide the necessary support, they have greater chances of presenting symptoms of burnout, anxiety, and severe depression. Medical education seeks for a balance between supervision and autonomy. When coming in contact with the first cases of a disease, it is expected that more supervision is needed to acquire technical knowledge. However, a supervisor may also be an important person to discuss challenging ethical situations, sharing the burden of tough decisions and helping the resident to gain experience and confidence [19].

A review to identify risk factors for *Sars-cov-2* infection among healthcare workers found that improper use or nonuse of PPE and certain exposures (intubation, direct contact with COVID-19 patients or contact with body secretions) are related to an increased risk of infection. They also found education and training, particularly information on how to properly use PPE, were consistently associated with a decreased risk of contamination [20]. Consistent with these findings, our study found that residents who felt prepared to treat COVID-19 patients exhibited a reduced odds of developing symptoms of anxiety and depression. Therefore, preparing residents is essential to prevent mental distress among them, especially if they will be working in direct contact with these patients or in a wing with high risk of contamination. Moreover, the same rational indicates that preparing armed forces personnel for the battlefield reduces their risk of developing posttraumatic stress disorder (PTSD) [21].

### Universities and teaching hospitals

This paper finds that for residents, the fear of transmitting COVID-19 to loved ones is associated to poorer mental health outcomes for measures examined. A recent study suggested that hospitals should consider providing accommodations and quarantine facilities for staff. This may be a feasible solution to help mitigate the negative psychological detriments for medical residents. Whether housing options provided to residents by their institution during the pandemic period protects them from presenting mental health symptoms is a matter for future studies [22]. Another measure hospitals could implement to minimize mental health distress in residents is providing clear procedure guidelines for COVID-19 treatment as well as the support of consultation committees for unforeseen ethical decisions. Institutions must be held responsible for creating a safe learning environment for medical residents where doubts, conflicts and uncertainties will be able to appear and dealt with as a group [23].

### Preventive psychological support and risk identification

Given that avoidance of the event is a core symptom of trauma, it highlights the importance of giving attention to physicians who avoid contact with COVID-19 patients [24]. This investigation found that residents with avoidance behavior were more likely to develop symptoms of anxiety, depression, and burnout. A study done with United Kingdom military peacekeepers on return from deployment showed that less psychological distress occurred in those who talked about their experience [25]. The authors suggest supervisors and peers pay attention to residents with signs of risky behavior, such as not attending meetings, “forgetting” about a case discussion or saying they are too busy.

Residents in contact with COVID-19 patients were significantly more afraid of maintaining habits of excessive disinfection after the pandemic. The threshold between healthy disinfection behaviors and obsessive-compulsive symptoms (OCS) might be unclear. A Canadian study reported an increase in OCS in the general population due to the fear of contamination by COVID-19. Individuals with the onset of OCS during the pandemic exhibit a ten to thirty-fold increase in presenting distress, generalized anxiety disorder and major depressive disorder [26]. In addition, residents in contact with COVID-19 patients were also more likely to present physical reactions. These symptoms such as sweating, difficulty breathing, nausea or flutter may be psychosomatic reactions, but a differential diagnosis with anxiety and depression must be made. Supervisors and peers should also be attentive to the onset of these symptoms among each other and refer these colleagues to treatment as early as possible. This attentive environment should also be reassuring and welcoming, so residents feel safe and confident that they will experience the empathy of their colleagues.

A review on the psychological impact of quarantine indicates that quarantined healthcare workers had more severe symptoms of posttraumatic stress than the general population. Given that healthcare workers experience more stigma than the general population, healthcare workers also presented more avoidance behaviors after quarantine, had a greater loss in income and were consistently more affected psychologically [27]. Residents that reported impaired personal relationships since the pandemic exhibited greater odds of developing burnout, anxiety, and depressive symptoms. Resident support groups are a powerful resource to prevent these conditions, and a larger support network, including friends and family of medical residents, who may help them by keeping in touch through online resources, such as video calls, is also important.

Although residents represent only a fraction of healthcare workers, their participation in the COVID-19 frontline is evident. The data in this study were collected during the first months of the pandemic, measuring the initial association of COVID-19 pandemic and mental distress, with alarming numbers that support the urgent need for intervention. This study suggests viable intervention strategies that can help residents during the COVID-19 pandemic and prepare them to deal with highly likely future pandemics.

The following limitations must be noted. First, this was a cross-sectional analysis involving residents exclusively from Brazil during the pandemic scenario, but we must consider that previous data on Brazilian medical residents’ mental health are similar to data found in other countries. Second, given the study’s cross-sectional design, we can state association but not causality. A convenient sample was used. This usually means that there is insufficient power to identify differences between population subgroups and can lead to several biases. Additionally, only 10% of medical residents were sampled, so generalizability may be limited. There is the possibility of a representation bias. Considering that the responses were voluntary, the participants may have been the most affected or those most willing to contribute. Therefore, it can be said that a relationship exists between the mental suffering presented by residents of Brazil and the pandemic scenario of COVID-19. Although the COVID-19 Impact Questionnaire is not validated, it was created by a multidisciplinary team with professionals working in the COVID-19 scenario and based on previous studies in other pandemics.

## Conclusion

This investigation found a high number of medical residents dealing with COVID-19 who have mental health symptoms, which reinforces the need of early identification and intervention to prevent the worsening of these conditions. These results suggest an increase in depression and anxiety symptoms among medical residents dealing with COVID-19, upstaging previous concerns about medical residents’ mental health. The prevalence of burnout is similar to that of a nonpandemic scenario.

Healthcare professionals need to be briefed about the risk and protective factors found in this and other studies to take better care of themselves and to identify peers at risk. Considering the severity of the pandemic scenario and the overburden of healthcare services, medical residents’ mental health must be at the frontline.

## Supporting information

S1 Data (XLSX)
